# Thin blue lines: product placement and the drama of pregnancy testing in British cinema and television

**DOI:** 10.1017/S0007087417000619

**Published:** 2017-09

**Authors:** JESSE OLSZYNKO-GRYN

**Affiliations:** * Department of History and Philosophy of Science, University of Cambridge, Free School Lane, Cambridge, CB2 3RH, UK. Email: jo312@cam.ac.uk.

## Abstract

This article uses the case of pregnancy testing in Britain to investigate the process whereby new and often controversial reproductive technologies are made visible and normalized in mainstream entertainment media. It shows how in the 1980s and 1990s the then nascent product placement industry was instrumental in embedding pregnancy testing in British cinema and television's dramatic productions. In this period, the pregnancy-test close-up became a conventional trope and the thin blue lines associated with Unilever's Clearblue rose to prominence in mainstream consumer culture. This article investigates the aestheticization of pregnancy testing and shows how increasingly visible public concerns about ‘schoolgirl mums’, abortion and the biological clock, dramatized on the big and small screen, propelled the commercial rise of Clearblue. It argues that the Clearblue close-up ambiguously concealed as much as it revealed; abstraction, ambiguity and flexibility were its keys to success.

Unilever first marketed the leading Clearblue brand of home pregnancy test in the mid-1980s. Since then home pregnancy tests have become a ubiquitous and highly familiar reproductive technology and diagnostic tool. Though also displayed in women's magazines, online and on pharmacy shelves, the plastic prognostic sticks are nowhere more strikingly and memorably advertised than when used as props in film and television drama. A particularly versatile trope, pregnancy testing can be accommodated in a range of narratives, including those about teenage pregnancy, single motherhood, romantic love, infertility, abortion or some combination thereof. Frequently portrayed on screen, a typical sequence of shots will link the self-tester's intent downward gaze to a close-up of the small, hand-held object, and a reaction shot of her delighted, disappointed or distraught face. Where did this highly stylized aesthetic convention come from and what can it teach us about the contribution of factors such as product placement to changing mores concerning the public visibility of reproduction?

Considering the present-day visibility of pregnancy testing, surprisingly little is known about its history.^1^ For the US, historian Sarah A. Leavitt has interpreted the ‘regular appearance’ of home tests in Hollywood cinema and on American television as a ‘sure sign’ that they have ‘entered the public consciousness’.^2^ Leavitt surveys plots and explicates the narrative function of pregnancy testing but does little to historicize the entertainment industry that produced dramatic scenes and storylines for public consumption. Lauren Rosewarne, in her investigation of menstruation in film and television, suggests that taboos against openly discussing or showing menstrual blood contributed to the rise of the visually innocuous plastic stick.^3^ And cultural-branding strategists have detailed the process whereby they aligned a 2006 television advertising campaign for Clearblue Digital with ‘third-wave’ ‘body-positive’ feminism by frankly showing urine for the first time.^4^ But we still lack anything resembling a historical account of how the on-screen trope of pregnancy testing became conventional or how it contributed to the normalization of pregnancy testing and, more generally, to a changing and increasingly public culture of reproduction.

This article will attempt to show how the convergent aesthetic conventions of televisual and cinematic pregnancy testing took shape and became constitutive of Britain's increasingly public culture of reproduction in the late 1980s and early 1990s. The politically tumultuous 1980s, a little-studied decade, saw an intensification of public interest in issues around human reproduction.^5^ From the highly publicized birth in 1978 of Louise Brown, the first ‘test-tube baby’, to the Human Fertilisation and Embryology Act 1990, the tabloid press, parliamentary debate and lobbying focused public scrutiny on IVF.^6^ Margaret Thatcher's Conservative Party, elected in 1979, emphasized ‘family values’; a series of private member's bills and the fledgling anti-abortion movement challenged various aspects of legal abortion; Victoria Gillick, a devoutly Catholic mother of ten, campaigned against the provision of contraception to minors; Mary Whitehouse railed against sex on television; and New Labour renewed the government's commitment to tackling ‘teenage pregnancy’ in the early 1990s.^7^ Meanwhile, unprecedentedly expensive and sophisticated marketing campaigns competed to stock pharmacy shelves with rival home pregnancy and ovulation test kits, aimed at a newly defined category of consumer: the older career woman trying to conceive.^8^

Products of a British boom in entrepreneurial biotechnology that coincided with NHS cutbacks and the rise in health consumerism, home pregnancy tests rapidly emerged as the most lucrative sector of the commercial diagnostics industry.^9^ In 1990, women in England and Wales were responsible for buying an estimated 4.8 million pregnancy tests, or around seven for every live birth.^10^ Competition between Clearblue (Unipath), Discover (Carter Wallace), First Response (Tambrands) and Predictor (Chefaro) resulted in advertisements in women's magazines and, with help from Britain's nascent product-placement industry, in television programmes and films shown in cinemas.

British cinema and television became unusually closely related in the 1980s, when Channel 4 (the fourth terrestrial channel after BBC1, BBC2 and ITV) quickly rose to prominence as a major player in the film industry.^11^ This article builds on research going back to Raymond Williams's *Television: Technology and Cultural Form* (1974), the classic ‘television studies’ book that also discussed cinema.^12^ It does so by using the pregnancy-test scene to investigate British cinema and television's shared technological resources, genre preoccupations and aesthetic sensibilities. Along the way, it extends film theory's renewed interest in the close-up by exploring the conventionalized sequence of shots that typically link a close-up of the tester's expressive face with that of a small, but dramatically charged, hand-held object – the home pregnancy test.^13^ Based on primary research in disparate paper, video and digital archives, it begins by setting the scene leading up to 1971, when Predictor, Britain's first home pregnancy test, debuted.

## Laboratories, toads and Predictor

The Aschheim–Zondek test, invented in Berlin in the late 1920s, was the first laboratory pregnancy test to become widely adopted in Britain. It involved injecting women's urine into female mice or rabbits and then dissecting the animals to inspect the ovaries for visible, physiological changes caused by the ‘pregnancy hormone’ today known as hCG (human chorionic gonadotrophin). Gory illustrations of the ‘bioassay’ took their place in journal articles and medical textbooks alongside schematic depictions of gynaecological examination.^14^ But beyond highly specialized publications, visualizations were scarce. Laboratories dealt only with doctors and kept a low profile at a time when animal research was frowned on and pregnancy testing was a ‘thoroughly unmentionable subject’.^15^

**Figure 1.**
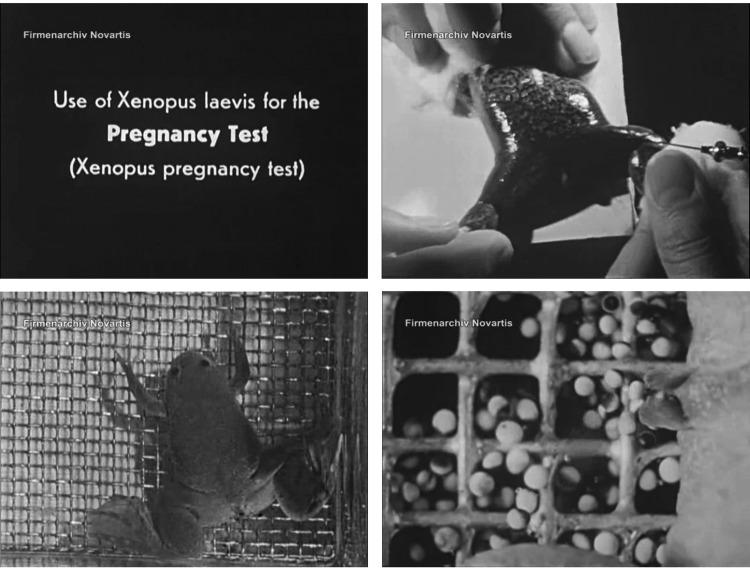
Screen captures from *The South African Clawed Toad* (Rolf Meier, *c*.1946) showing (from top left to bottom right): title card, urine injection, test toad and an ‘extremely positive reaction’. The wire mesh, clearly visible in the bottom left image, was used in some laboratories to prevent the toad from ruining the test by devouring her own eggs. Copyright Novartis International Ltd.

The adoption by the NHS of *Xenopus laevis* – a toad from South Africa that laid large, visible eggs when injected with human pregnancy urine and so did not have to be dissected in the course of a test – increased the efficiency, cost-effectiveness and social acceptability of pregnancy diagnosis after the Second World War.^16^ It was around this time that the first silent 16 mm films of laboratory pregnancy testing began to circulate. In the late 1940s, Brian Stanford, a London doctor and medical filmmaker, produced *The Aschheim–Zondek Test for Pregnancy* (in colour) and the pharmaceutical laboratories of the Basel-based chemical company Ciba produced *The South African Clawed Toad* (in black and white), which covered various aspects of the life cycle of *Xenopus*, as well as its use in human pregnancy testing (Figure 1).^17^

Both films were made for doctors by doctors. The general public remained more or less in the dark about the use of animals in pregnancy testing.^18^ Women's magazines and pregnancy advice manuals occasionally mentioned bioassays in the 1930s and 1940s, but typically discouraged pregnancy testing as an expensive luxury.^19^ Only in 1961 did the popular magazine *Woman* endorse the toad test as a ‘modern scientific achievement’, and then only in response to concerns about Primodos, a controversial pregnancy-test drug that induced uterine bleeding and was suspected of causing miscarriages and birth defects.^20^ Medical discretion gave way to open public debate in 1965, when commercial laboratories began advertising directly to women not as patients, but as consumers.^21^ By then, mass-produced immunological test kits had supplanted living animals and a ‘huge shift’ in vocabulary had occurred. Previously unmentionable words such as ‘pregnancy’, ‘abortion’ and ‘termination’ were more frequently printed in the press, novels and private letters.^22^ Pregnancy testing, though generally missing from histories of the ‘sexual revolution’, was hotly debated in newspapers and magazines alongside the closely related issues of contraception and abortion.^23^

In 1965, laboratories began taking out classified adverts for their diagnostic services. The standard fee of two pounds a test was equivalent to about a week's rent in Leeds in 1968.^24^ Belmont Laboratories, the largest and most enterprising, fuelled the controversy by placing posters in British Rail and London Underground stations. Some doctors complained, but progressive journalists increasingly portrayed the medical profession as paternalistic and out of step with public opinion. Novelists including Lynne Reid Banks, Anthony Burgess, David Lodge and Andrea Newman narrated pregnancy testing in the 1960s.^25^ By the end of the decade, pregnancy testing confronted not only readers, but also radio listeners, television viewers and cinema-goers.

**Figure 2.**
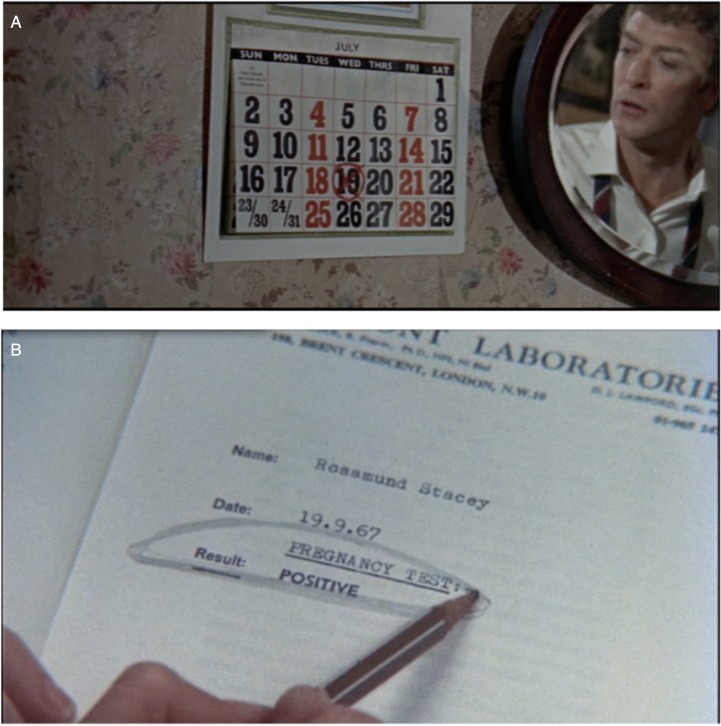
Screen captures from (A) *Alfie* (Lewis Gilbert, 1966), produced by Lewis Gilbert and Sheldrake Films, distributed by the BFI; (B) *A Touch of Love* (Waris Hussein, 1969), produced by Amicus and Palomar, courtesy of STUDIOCANAL Films Ltd.

A 1960s ‘media revolution’ made sex and reproduction more publicly visible than ever before.^26^ British ‘kitchen sink’ cinema contributed to it by audaciously confronting audiences with challenging issues including juvenile delinquency, homosexuality, teenage pregnancy, mixed-race relationships, unmarried motherhood and illegal ‘backstreet’ abortion.^27^ A missed menstrual period, morning sickness or quickening typically confirmed pregnancy in these films. Often the pregnant character was a young, working-class woman who could not have afforded a test anyway. In Tony Richardson's adaptation of Shelagh Delaney's 1958 play *A Taste of Honey* (1961), pregnant schoolgirl Jo (Rita Tushingham) is exhilarated when she feels her baby's first kick as a thunderstorm is brewing. The philandering eponymous antihero (Michael Caine) of Bill Naughton's *Alfie* (1966) is alerted by a calendar to his girlfriend's condition (Figure 2A). And pregnancy is disclosed when a factory girl runs to the lavatory to be sick in *Up the Junction* (1968). Only Waris Hussein's *A Touch of Love* (1969), based on Margaret Drabble's novel *The Millstone* (1965), portrayed a ‘positive’ pregnancy test result (Figure 2B).

The opening sequence of *A Touch of Love*, also known as *Thank You All Very Much*, shows the decidedly middle-class lead, Rosamund Stacey (Sandy Dennis), restlessly circling her lab report in the British Museum reading room, where she is unable to concentrate on her doctoral thesis. The full name of the lab falls outside the frame, but the visible address and postcode identify its provenance as Belmont Laboratories. The earliest cinematic pregnancy test I have seen, the report was probably ‘chosen by a diligent prop man who had done his research and used an actual address’.^28^ Less a trendsetter than an early outlier, Rosamund's lab report is a testament to the degree of publicity stirred up by Belmont and other diagnostic services in the late 1960s.

On BBC1's evening news magazine programme *24 Hours* (10 September 1969) the director of Belmont Laboratories explained that it was ‘the woman's prerogative to know if she was pregnant as quickly as possible’.^29^ The BBC1 documentary *Mother's Pride* (12 February 1971) linked pregnancy testing to a woman's decision to have an abortion, while ‘Congratulations it's a toad’ (4 April 1971), a cheeky episode of London Weekend Television's medical sitcom *Doctors at Large*, made light of the use of toads and urine for human pregnancy diagnosis. On BBC Radio 2, *Woman's Hour* (6 March 1970) investigated commercial laboratories, while on Radio 4 *You and Yours* (13 January 1972) reported on the first self-testing kits.^30^

By the time Chefaro, a subsidiary of the Dutch pharmaceutical company Organon, launched Predictor in 1971, Belmont and other labs had already cultivated a sizable market. Research commissioned by Chefaro found that two-thirds of women aged fifteen to forty-four had already heard of laboratory tests for pregnancy and 62 per cent agreed with the statement ‘It is an excellent method for checking up before going to the doctor’.^31^ Predictor initially retailed for £1.75, just under the going rate for a laboratory test, and was supported by a £60,000 advertising campaign aimed directly at consumers.^32^ An advertisement in the free London magazine *Girl about Town* (14 June 1976) compared the ‘pregnancy test you do yourself’ to the ease of ‘buying a lipstick’. But the consumer watchdog organization *Which?* warned that ‘clumsy’ users ‘could get a wrong result, and waste almost £2’; many women continued to rely on doctors, family-planning clinics, chemists and women's centres.^33^ Whereas advice literature before the 1960s discouraged laboratory pregnancy testing, *Which?* endorsed diagnostic services. In deference to medical authority, however, it stopped short of recommending home test kits. In 1982 *Which?* described Predictor (now £4.75) and its sole competitor, Carter Wallace's Discover-2 (£5.25), as ‘fiddly to use’.^34^

Women's afternoon television programming frankly discussed pregnancy and childbirth in the 1950s.^35^ Dramas, however, typically elided the process of realization or discovery. For instance, in the very first series of *Coronation Street* (1960), Linda simply tells her husband that she is expecting, while unabashedly smoking a cigarette.^36^ Some sixteen years and more than 1,500 episodes later, the classic ITV serial had the Langtons anxiously waiting for a doctor to communicate the result of Deirdre's pregnancy test.^37^ In the 1980s, Penny (Jan Francis) and Vince (Paul Nicholas) spend an entire episode of the popular sitcom *Just Good Friends* (BBC1, 1984) contemplating the future while waiting for a doctor to divulge the result of Penny's pregnancy test.^38^ Parents discuss their teenage daughter's lab report over breakfast in the bilingual sketch comedy programme *Let's Parlez Franglais* (Channel 4, 1984). Rehana (Meera Syal) advises her friend to ‘see a doctor and get a pregnancy test’ in *Majdhar* (Channel 4, 1985), a film about immigration and arranged marriage. And the comedy serial *The Two of Us* (ITV, 1990) ends with the unmarried Elaine Walker (Janet Dibley) telephoning her doctor about a pregnancy test, the result of which is never disclosed to viewers.

**Figure 3.**
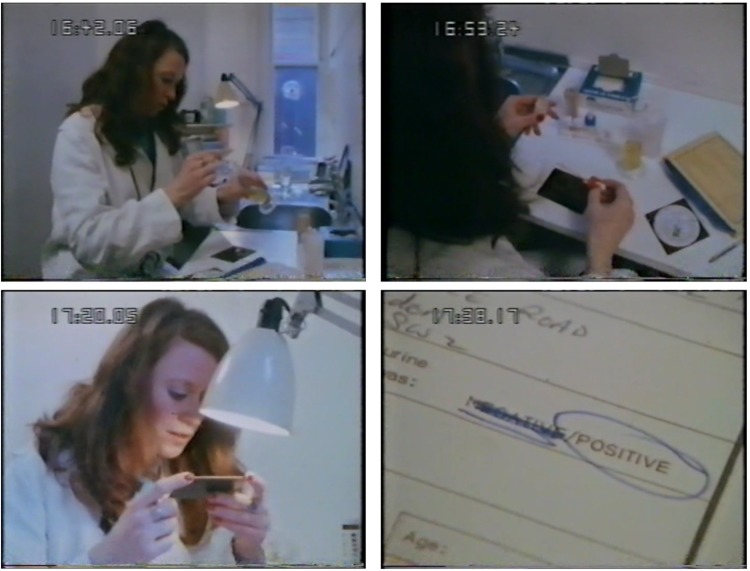
Screen captures from *The London Programme*, Production Number 22561, recorded on 13 April 1978. Presented by Godfrey Hodgson, produced by Barry Cox, report by Greg Dyke.

Beyond drama and comedy, non-fiction television occasionally went behind the scenes to show laboratory pregnancy testing in action.^39^ *The London Programme* (LWT, 1978) reused old footage from Ciba's *Xenopus* film and showed the new rapid slide test in action in a documentary about Primodos (Figure 3).^40^ Peter Williams's *Test Tube Explosion* (ITV, 1982), timed to coincide with the start of the high-profile public ‘Warnock inquiry’ into IVF and human-embryo research, linked pregnancy testing to a treatment regime that left patients impatiently waiting by the phone to see if they would ‘hold on to their pregnancies’.^41^ And in 1987, an ITV news report on the Alton Bill, which aimed to curb late abortions, showed pregnancy testing by the British Pregnancy Advisory Service (BPAS), a leading abortion provider.^42^ From the mid-1980s, however, serialized drama became the main vehicle for beaming pregnancy tests into British homes.

## Socially engaged drama and realism on television

Television dramas in the 1960s and 1970s were typically shot on video in a studio, where it was common to change the composition of the shot by physically moving the (cumbersome and heavy) camera closer to or further from the action, rather than by cutting in post-production. Tight production schedules and budgetary constraints left little time or money for experimentation, and most television dramas followed the standard visual ‘grammar’ of wide shots of set and closer shots of main characters, alternating with close-ups in a very conventionalized system.^43^ Then, in the early 1980s, a new kind of evening soap opera pioneered by *Brookside* and *EastEnders* created new narrative and aesthetic possibilities – including for pregnancy testing.

Commissioned from Mersey Television, a small, independent production company run by Phil Redmond, Channel 4's *Brookside* was filmed not in a studio, but on location at a suburban housing estate on the outskirts of Liverpool.^44^ Redmond, then known for the BBC1 children's drama *Grange Hill*, innovatively used Sony's ‘complete “glass to glass” production system’ to produce *Brookside* at a ‘cost-base the new Channel 4 could afford’.^45^ Each house was fitted with concealed electric cables and cameras, enabling more ‘realistic’ action than possible on a set, and Redmond's adoption of cinema's highly mobile Steadicam further liberated *Brookside* directors from constraints imposed by the traditional and unwieldy television camera system of dolly and tracks.^46^

Cinematic realism extended to other, more controversial aspects of the show. Whereas *Coronation Street* had become a cosily nostalgic reflection of the past, *Brookside* boldly addressed topical social issues from mass unemployment, strikes and deindustrialization to homosexuality, AIDS and the privatization of health care. Its diverse characters included not only trade unionists, but also aspirational yuppies, Channel 4's target audience. The premiere, watched by 7 million on first transmission (2 November 1982), stirred up controversy over bad language. Television's ‘moral watchdog’, Mary Whitehouse, was soon calling for the resignation of chief executive Jeremy Isaacs, but influential *Daily Mirror* critic Hilary Kingsley was hooked from start.^47^ *Brookside* did not challenge the viewing figures of *Coronation Street*, but immediately became Channel 4's most popular programme.^48^

The BBC, having fallen behind ITV in the ratings, decided to produce its own edgy, realist soap along the lines of *Brookside*. *EastEnders*, first broadcast on 19 February 1985, surpassed expectations to rapidly become Britain's most popular television programme, reaching an audience of 23 million in February 1986.^49^ It targeted not only women, but also men and younger viewers. Prominent teenage characters experienced credible ‘teenage problems’ from unemployment to abuse and pregnancy.^50^ Whitehouse accused the BBC of corrupting ‘family viewing time’ with gratuitous sex and violence, but the salacious storylines, especially that of sixteen-year-old Michelle Fowler's pregnancy, captivated millions.^51^ Having grown up on a North London council estate, *Grange Hill* actress Susan Tully brought credibility to the role of Michelle. And in an unusual, perhaps unique, configuration, it was Michelle's grandmother, Lou Beale (Anna Wing), who instigated her home pregnancy test.

First broadcast at 7 p.m. on Tuesday, 17 September 1985, the pregnancy-test episode begins with a cup of tea. Lou, the tough but benevolent matriarch in whom Michelle has confided, advises her to ‘only have one cup’ to keep her from ‘running up and down to the lav’. Lou then explains that she is ‘going up the chemist's’ to ‘buy one of them kits’ because she wants to know if Michelle is pregnant ‘for definite’. Michelle readily accepts Lou's conspiratorial plan, which involves lying to her mother about staying home from school and doing the test later in the day when they will have the place to themselves. When Lou returns from the chemist's, the coast is clear and she hands Michelle the test.

Packaging visibly exchanges hands between Lou and Michelle, but the test itself is not shown; viewers are left to infer that Michelle has successfully performed the test in the privacy of the household lavatory. Whereas *Which?* described lay users as ‘clumsy’ and home test kits as ‘fiddly’, television typically implied an effortless procedure, off screen and between scenes. The brand, in the case of *EastEnders*, is not clearly visible, but resembles Predictor. When Michelle emerges, having done the test, she must ‘wait thirty minutes’, an ordeal that ‘calls for [another] cup of tea’. An off-screen clock ticks audibly and ominously, building the tension. Midway through the episode, Michelle emerges from the bathroom for a second time. ‘It's positive’, she announces. The episode, but not the storyline, ends with a cliffhanger as Lou asks, ‘Who did it, Chelle? Who's the father?’

Tabloids and viewers speculated that Michelle would miscarry, possibly in a motorcycle accident, but the titillating question of paternity generated by far the most publicity and discussion.^52^ Jonathan Powell, then head of drama at the BBC, claimed that the ‘human story’ had ‘touched a public nerve’.^53^ A review in *The Listener* attributed the runaway success of *EastEnders* to the ‘great drama of Michelle Fowler's schoolgirl pregnancy’.^54^ On Christmas Day 1986, 30 million watched as Michelle's mother learned the identity of the baby's father. The ‘whodunnit’ narrative not only hooked viewers, but also allowed the BBC to fulfil its public-service duty by creating plausible contexts for a health visitor, home tutor and social worker didactically to inform Michelle – and viewers – about the ‘rights and entitlements’ of young, unmarried mothers.^55^ Teachers used the serial to frame classroom discussions, and even Whitehouse approved of Michelle's decision against abortion as a ‘very positive storyline’.^56^ Kingsley lauded *EastEnders* for ‘teaching Britain's kids more about you-know-what than prissy sex manuals’, even reporting that it had disabused a sixteen-year-old boy of the mistaken belief that pregnancy tests ‘give you abortions’.^57^

Michelle Fowler was at the vanguard of a trend that made pregnancy a controversial staple of 1980s British television well beyond drama. Following the ruling against Victoria Gillick, actress Susan Tully promoted the Family Planning Association (FPA) to minors in BBC2's educational documentary ‘Too young to have a baby?’ (12 March 1986). Anne Diamond became Britain's first visibly pregnant television presenter on ITV's *Good Morning Britain* (1987).^58^ And Sweden's Neneh Cherry sparked a British debate when she performed her hit single ‘Buffalo stance’ on BBC1's *Top of the Pops* while seven months pregnant (22 December 1988). Documentaries investigated ‘schoolgirl mums’ and IVF while an impressive range of fiction genres appropriated the home pregnancy test for a variety of ends.^59^

Zenith Productions’ *The Dead of Jericho* (ITV, 1987), the first television adaptation of an Inspector Morse novel, featured a ‘positive’ Predictor test kit in the medicine cabinet of a distraught Ms Anne Stavely (Gemma Jones).^60^ A close-up of a generic box resembling that of Predictor sets the scene for role-reversing male pregnancy in the ‘Parallel universe’ episode of the cult science fiction sitcom *Red Dwarf* (BBC2, 1988). In ‘Women's trouble’, one of the first episodes of the long-running sitcom *Birds of a Feather* (BBC1, 1989), sisters Tracey (Linda Robson) and Sharon (Pauline Quirke) discuss contraception, infertility and STIs in the lavatory while Tracey brushes her teeth and Sharon waits for the result of her Predictor. And in ‘The waiting game’ (Channel 4, 1991), an episode of Guyanese British sitcom *Desmond's*, a ‘negative’ pregnancy test is passed around like a hot potato until it ends up in the hands of a befuddled Desmond Ambrose (Norman Beaton). All these broadcasts showed packaging or the test result, but not the diagnostic process itself.

Only Peter Bebbington's ‘Positively negative’ (Channel 4, 1990) – a short teleplay in youth editor Stephen Garrett's *He-Play*/*She-Play* series to showcase new talent – depicted most of the procedure. Shot in a single day, ‘Positively negative’ is essentially a compressed ‘kitchen sink’ drama about a young Yorkshire couple waiting for the result of a pregnancy test. It opens with a close-up of a First Response box and proceeds to show every step of the process with dynamic close-ups of hands, urine and test kit. Though not apparently intended as didactic, the drama might as well be an instructional video for First Response. Marketed by Tambrands, the US company best known for Tampax, First Response retailed in Britain for £6.95. The test required the user ‘to collect a urine sample’, ‘add it by dropper to a test tube containing powder’, swirl the tube ‘to mix the powder with the urine’, and then to leave it standing ‘for five minutes’ before finally pouring the ‘contents of the test tube’ ‘into a small well covered with a filter paper’. The appearance of a ‘clearly visible’ pink colour indicated a positive result; no colour meant negative.^61^

Though her urine is already collected in a plastic receptacle at the start of the play, Tracey (Victoria O'Keefe) performs every other step of the test on screen as boyfriend Keith (Chris Garner) encourages her to ‘bung it in’, stumbles over technical jargon while reading the instructions and confirms the definitely pink, ‘positive’ result.^62^ An unusually detailed portrayal of pregnancy testing, ‘Positively negative’ captured an elaborate ritual on the verge of extinction. In the early 1990s, televisual and cinematic pregnancy testers overwhelmingly switched to Clearblue, the first brand that viewers could be counted on to recognize.

## The first Clearblue close-ups

Launched in June 1985 by Unipath, a subsidiary of the Anglo-Dutch company Unilever, Clearblue was a game changer. Marketing literature boasted that its ‘advanced technology’ made the kit ‘less messy’ and ‘more accurate at an earlier stage’, and gave a ‘clearer result’ in ‘less time’.^63^ The rapid commercial success of Clearblue had as much to do with slick advertising and design as it did with its innovative use of monoclonal antibodies. Beyond technological sophistication, it came in ‘altogether a more attractive package for women – less daunting to use and coupled with advertising designed to give it more feminine appeal’. Market research indicated that the brand name, distinctive fan-shaped logo and blue colour carried a ‘positive meaning’. As with all other home pregnancy tests, Clearblue was marketed to women hoping to conceive, ‘so red for danger would have been quite inappropriate’.^64^

Unilever correctly anticipated that the British market, already valued in 1985 at £3 million, or 600,000 tests a year, would expand as public-sector cutbacks pressured doctors to perform tests only when medically needed. As an article in *The Guardian* warned, the next time a woman asks her doctor for a pregnancy test, she could be told, ‘I'm sorry, but the National Health Service can't afford to find out if you're pregnant. You'll have to go to the chemist and buy a kit’.^65^ Beyond cuts, Unipath cited the ‘general heightening of health awareness, which means many women are keen to know whether they are pregnant or not as soon as possible so that they can adjust their lifestyle accordingly – another reason for the market to expand’.^66^ As Unipath's marketing director told the *Financial Times*, ‘It is very important to know as early as possible that you are pregnant so that you can change your lifestyle – for example stop smoking and drinking’.^67^

Clearblue captured 30 per cent of the British market in just three months. It was followed in April 1987 by Clearplan, an ovulation test, and in July 1988 by Clearblue One Step.^68^ Winner of the 1989 British Design Award in Medical Equipment, Clearblue One Step innovatively dispensed with the messy handling of liquid reagents and introduced the now-familiar plastic stick with an absorbent wick at one end and two display windows at the other.^69^ At £8.35, it cost slightly more than First Response.^70^ By then, Unipath had captured half the British market, which had more than doubled to £7 million and was still growing at a rate of 15 per cent every year. Global sales approached £100 million annually.^71^ Billed as ‘the most advanced pregnancy testing kit in the world’, the new product, known as Clearblue Easy in the US, incorporated the use of monoclonal antibodies in a ‘novel format derived from a unique rapid assay technology developed solely by Unipath and protected by patents’.^72^ Advertisements in women's magazines typically portrayed white, middle-class women serenely waiting for the clear blue result in the comfort of their own softly lit bathroom. But the earliest on-screen users were pregnant schoolgirls determined to abort and British Asian women caught between cultures and in difficult relationships.

British Asian cinema took off in the late 1980s with Hanif Kureishi's *My Beautiful Laundrette* (1985) and Gurinder Chadha's *I'm British But …* (1989), films both supported by Channel 4. The former, though made for television on a low budget in six weeks, was theatrically released and critically acclaimed. The latter launched Chadha's stellar career. Following Channel 4's lead, the BBC also began to support young British Asian talent. In the early 1990s, the corporation hired Meera Syal, freshly graduated in English and drama from Manchester University, to write the screenplay for a feature-length drama for *Screen Two* (BBC2, 1985–1994), the successor to *Play for Today* (1970–1984).^73^ She came up with *My Sister Wife*.

First broadcast on 23 February 1992, *My Sister Wife* concerns the conflict between a wealthy Pakistani businessman's two wives: Farah Khan (Syal) and Maryam Shah (Shaheen Khan). At the start of the film, the British-born Farah is modern while the Asian-born Maryam is traditional; by the end, their roles have reversed and Asif Shah, the husband (Paul Bhattacharjee), is dead, accidentally poisoned by Farah's misfired plan to murder her rival. Along the way, sexuality, gender relations and reproductive politics are provocatively dramatized in relation to contraception, pregnancy, miscarriage and sex-selective abortion. A crucial moment in the rivalry between the sister-wives comes when Farah discovers a pack of Clearblue pregnancy tests in Maryam's dresser drawer. In a scene, the mood of which could not be more different from the serenity depicted in women's magazines, the discovery prompts Farah to plot revenge: ‘Kill one baby, start another. So you want to fight dirty, my sister-wife; I haven't even begun’.^74^ Though otherwise unusual in many respects, the sequence linking Farah's downward gaze to a Clearblue close-up (albeit of clearly branded packaging, not the stick itself), followed by a dramatic reaction shot, would soon become standard.

**Figure 4.**
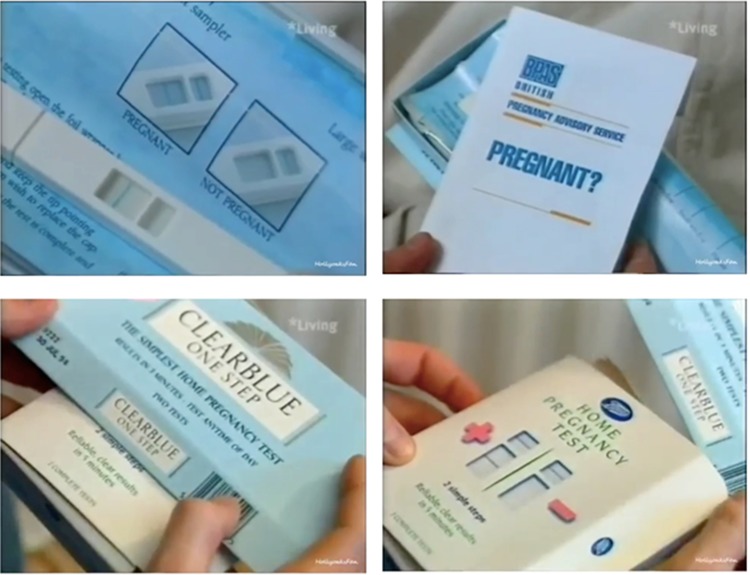
Screen captures from Channel 4's *Brookside*, first broadcast 14 December 1992, later rebroadcast on Living.

Meanwhile, on Channel 4, *Brookside* introduced multiple pregnancy tests into a schoolgirl-pregnancy-and-abortion storyline timed to coincide with Christmas, a favourite season for dramatic pregnancy testing. Fifteen-year-old Leanne Powell (Vickie Gates) first chooses the cheaper Boots model, which she uses in the toilet of her friend's house. Hoping for a negative, Leanne purchases several more tests in the next episode. A series of close-ups compares a positive ‘clear blue’ result with the instructions, plugs BPAS and shows Clearblue One Step and Boots packaging (Figure 4). Recognizable logos and legible text convey information as her friend Jacqui Dixon (Alex Fletcher) advises Leanne to go to BPAS. Jacqui didactically explains – as much to young viewers as to Leanne – that the service is confidential and ‘you don't have to be over sixteen to have an abortion’. With help from Jacqui's mother DD (Irene Marot), a devout Catholic who finds the hastily hidden tests under her daughter's bed, Leanne does have the pregnancy terminated shortly after her sixteenth birthday. Not a conclusion, the abortion drives the plot along as Leanne's parents find out about DD's meddling and turn against her.

On 21 January 1993, the day after Leanne's fictional abortion, *Bhaji on the Beach* opened in cinemas around the country. Directed by Chadha and scripted by Syal, it made history as the first feature film directed by an Asian woman in Britain. Co-produced by Channel 4, the film also brought the Clearblue close-up to the big screen. Syal, who had previously collaborated with Chadha on a short Channel 4 film about arranged marriage, came to her with the basic idea for *Bhaji*: a group of Asian women from Birmingham go to Blackpool for the day.^75^ In an interview with the British Film Institute's magazine *Sight & Sound*, Chadha explained that she ‘didn't want the film to be “just a comedy”’. Thus she ‘picked the two most taboo subjects within the Asian community – mixed relationships and separation and divorce – and threw them in as well’.^76^ It is in connection with a mixed relationship, that of the young student Hashida (Sarita Khajuria) and her Afro-Caribbean boyfriend Oliver (Mo Sesay), that pregnancy testing comes in.

**Figure 5.**
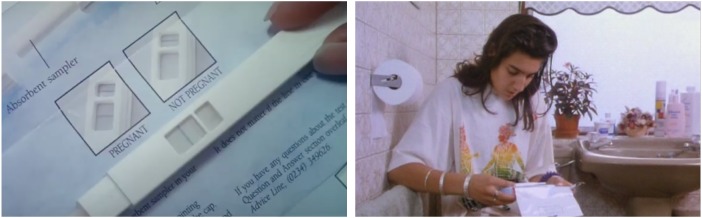
Consecutive screen captures from *Bhaji on the Beach* (Gurinder Chadha, 1993), produced by Channel 4 Films and Umbi films, distributed by Channel 4. The second ‘control’ line in the smaller box is not visible, possibly because a ‘negative’ test result was manipulated on set to give the impression of a ‘positive’ result. See also in Figure 4 (top left).

As with Farah in *My Sister Wife* and Leanne on *Brookside* (who also uses a Boots test kit), Hashida opts for Clearblue. In a similar sequence of shots, the camera cuts from a close-up of the ‘positive’ test result – Hashida’s point of view – to a reaction shot of her troubled, downward gaze; ‘Oh shit’, she exclaims (Figure 5). Though easily mistakable for ‘product placement’ – the controversial form of embedded marketing made famous a decade earlier when Steven Spielberg's *E.T.* (1982) conspicuously advertised Reese's Pieces – the use of Clearblue up to and including *Bhaji* was, as far as Syal remembers, ‘simply the props department getting the most easily available over-the-counter product at the time’.^77^ From 1994, however, product placement began to play a role.

## Embedded marketing

Britain's Independent Television Commission (ITC) prohibited production companies from accepting payment from advertisers, but allowed advertisers to pay an intermediary to supply their brand of a particular prop free of charge to production companies. Ever since the first British placement agency was founded in 1984, Britain's unique ‘free prop supply’ system has acted as a ‘de facto product placement industry’, supplying the non-commercial BBC, the ‘word's largest public service broadcaster’, and its commercial rivals with branded props.^78^ Founded by Graeme Atkinson in 1988, Prop Portfolio, a placement agency based in the Northamptonshire market town of Wellingborough, began working with Unipath in 1994 to ensure that more on-screen pregnancy testers chose Clearblue. Crucially, Unipath was ‘never … concerned with the morality of the scenario [and] as long as the test was seen working and being accurate they were happy’.^79^

Unipath's no-strings-attached policy meant that – in stark contrast to the happily married women portrayed in women's magazines – Clearblue was placed in the most scandalous televisual relationships. Prop Portfolio's first ever placement of Clearblue, on ITV's *Emmerdale* (1994), a soap set in the Yorkshire Dales, was in the hands of character Rachel Hughes (Glenda McKay), who had been carrying on an affair. Two years later, when Georgia Simpson (Helen Grace) used a pregnancy test as part of *Brookside*’s boundary-pushing incest storyline, she too chose Clearblue.

Product placement, though more commonly associated with cars, electronics, food, soft drinks and cigarettes, was a particularly effective means of marketing Clearblue in Britain.^80^ The BBC, which still controlled a significant market share, did not broadcast commercials, so viewers would see Clearblue only when it was embedded in a drama or comedy or, less frequently, shown on the news. On the commercial channels, marketing campaigns were prohibitively expensive and heavily regulated. Adverts for ‘sanitary products’, then only recently allowed on television before 8 p.m., were not permitted to show ‘product shots’ of tampons or pads. Nor could they visually demonstrate or describe how such products worked, mention ‘menstruation’ or show ‘anything remotely resembling blood’. Instead of blood, a blue liquid was used. The ITC's position on pregnancy testing was similarly restrictive. Chefaro, having missed out on placement, necessarily adopted an oblique approach when it launched a £1.2 million television ad campaign for Predictor in 1995. As a report in the trade journal *Community Pharmacy* put it,

Beautifully manicured hands twist the top off a fountain pen, remove the nib, then replace it. They whisk a mascara pen from its holder, then put it neatly back. That's how easy it is to use a pregnancy test, we're told, as a glob of blue splodges down over a pink sponge and a blonde sits gazing wistfully into the mid-distance.^81^

The television commercial echoed Chefaro's 1976 magazine advert in emphasizing ease of use, only substituting lipstick with mascara. But as one critic remarked, the scene was ‘a far cry from what goes on behind the closed doors of most real bathrooms, as women drag themselves out of bed to urinate on a stick at first light and find out whether a late period means what they think it means’. It was also a far cry from the realism provided though product placement. The company, she reported, ‘would have liked to use the word urine’, as it did in print advertising, but ‘simply [was not] allowed to on TV’. Though manufacturers generally agreed that television meant ‘a short-term surge in sales’, the cost was ‘prohibitively high’, and even Chefaro could not afford to ‘screen their ads in the ultra-expensive London region, despite the fact that it accounts for about 35 per cent of national sales’.^82^

Unipath generally preferred to advertise in women's magazines, with posters, and occasionally on the radio. But the company responded to Chefaro's television campaign by launching its own in November 1996.^83^ Where rival Chefaro had coped with ITC constraints by resorting to an extreme close-up of Predictor's pink spongy tip, Unipath invented ‘Molly’, an inoffensive cartoon character who also promoted Unipath's new, faster test in women's magazines. But television commercials remained opaque and product placement continued to play a major role; Prop Portfolio placed Clearblue in dozens of television and film productions every year in the late 1990s and early 2000s, including in a newly flourishing cinematic genre: the British romantic comedy.

## From romantic comedy to WombTube

The romantic comedy, a Hollywood staple previously unknown in British cinema, took off in Britain in the 1990s as a result of the combined efforts of screenwriter Richard Curtis, actor Hugh Grant and the independent production company Working Title. Originally made for *Film on Four*, Channel 4's answer to the BBC's *Screen One* and *Screen Two*, *Four Weddings and a Funeral* (1994) established a winning formula for films about middle-class, metropolitan characters. It was set in an enchanted, fairy-tale London, where romance blossomed between American and English lovers.^84^ Curtis and Grant followed the unexpected international box-office success of *Four Weddings*, their first collaboration with Working Title and the highest-grossing British film up to that point, with *Notting Hill* (1999), *Bridget Jones's Diary* (2001), *Love Actually* (2003) and *Bridget Jones: The Edge of Reason* (2004). American actresses speaking Estuary English and transatlantic relationships boosted the international appeal of British romantic comedies in the ‘Cool Britannia’ years of the Spice Girls, Britpop and *The Full Monty* (1997).^85^

Not only love and marriage, but also infertility and reproduction motivated plots. *Notting Hill* ends with a visibly pregnant Anna (Julia Roberts) reclining on a park bench with Will (Hugh Grant), and *Maybe Baby* (2000), adapted by Ben Elton from his semi-autobiographical *Inconceivable* (1999), ends with Sam (Hugh Laurie) and Lucy (Joely Richardson) still trying for a baby after a failed IVF cycle. In *Sliding Doors* (1997), an English ‘concept-comedy’ financed by the American production company Miramax,^86^ Helen (Gwyneth Paltrow) emerges from the toilet with a ‘positive’ pregnancy test in hand. Helen, who experiences a miscarriage in both parallel storylines of the narratively experimental film, chooses Predictor, the brand clearly visible on the packaging she holds up to show her friend and viewers. But most pregnancy testers in British romantic comedies used Clearblue.

**Figure 6.**
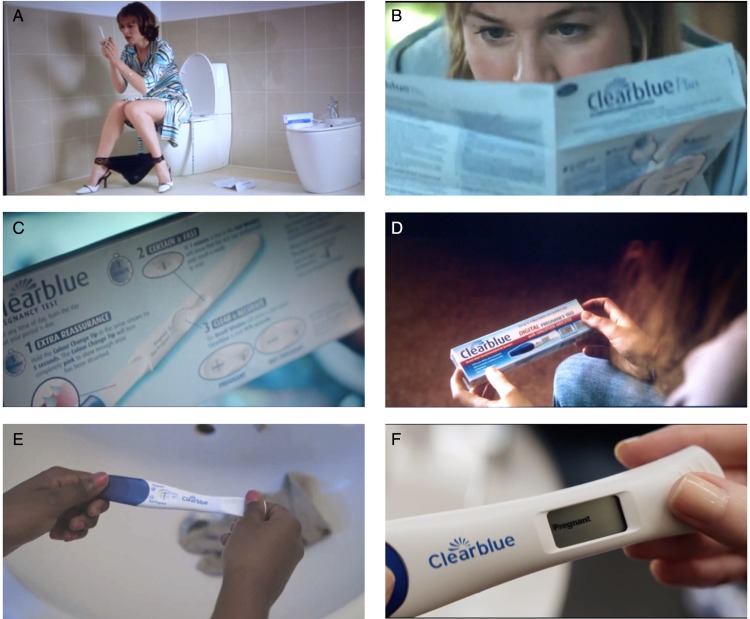
Screen captures from (A) *The One and Only* (Simon Cellan Jones, 2002), produced by Assassin Films, distributed by Pathé; (B) *Bridget Jones: The Edge of Reason* (Beeban Kidron, 2004), produced by Little Bird, STUDIOCANAL, Working Title; distributed by Universal and Miramax; (C) *Puffball* (Nicolas Roeg, 2007), produced by Amérique, Dan Films, Grand Pictures, Tall Stories; distributed by Verve; (D) *Albatross* (Niall MacCormick, 2011), produced by CinemaNX and Isle of Man Film, distributed by Entertainment One; (E) *Love Letter* (Lucia Yandoli, 2012), courtesy of Lucia Yandoli; (F) *Bridget Jones's Baby* (Sharon Maguire, 2016), produced by Miramax, Perfect World, STUDIOCANAL, Universal and Working Title; distributed by Universal.

**Figure 7.**
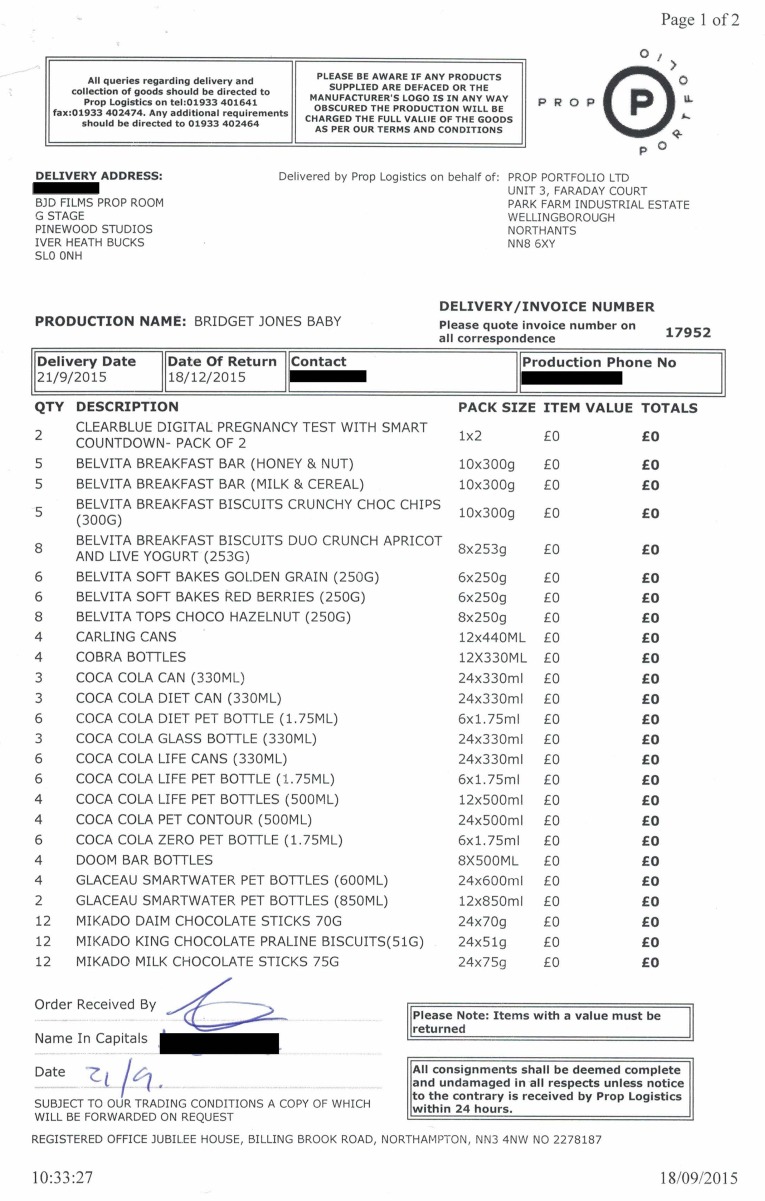
Standard delivery notice (21 September 2015) for *Brigit Jones's Baby*, listing two Clearblue Digital pregnancy test packs (of two), alongside Belvita breakfast bars, Coca Cola cans and bottles and various other brand-name products. Courtesy of Graeme Atkinson and Scott Shearsmith of Prop Portfolio Ltd.

In *The One and Only* (2002), a box of Clearblue rests on a bidet in the bathroom of an Italian footballer's Newcastle mansion as his English ‘trophy wife’ Stevie (Justine Waddell) counts down the seconds (Figure 6A). When the doorbell interrupts her counting, she rushes downstairs, pregnancy test in mouth, only to be congratulated by the kitchen fitter Neil (Richard Roxburgh). The result is positive and it is love at first sight. Clearblue achieved greater prominence in *Bridget Jones: The Edge of Reason* (2004), a film often singled out by critics for its flagrant product placement. Following close-ups of an open box in profile and of the heroine (Renée Zellweger) intently reading instructions, the test is then interposed between Bridget and Mark Darcy (Colin Firth), embodying the potentialities of their future together as they discuss children (Figure 6B).^87^ The result is negative. But twelve years later, when Zellweger and Firth reprised their roles in *Bridget Jones's Baby* (2016), the result – of a Clearblue Digital – is ‘Pregnant’ (Figure 6 F; Figure 7).

Beyond romantic comedy, a range of genres appropriated the Clearblue close-up in the early twenty-first century. In *Puffball* (2007), a supernatural drama based on the Fay Weldon novel from 1980, Liffey (Kelly Reilly), an ambitious young architect, downs a glass of red wine while nervously waiting – stopwatch in one hand and cigarette in the other – for the result of her pregnancy test. The Clearblue logo is conspicuous in close-ups of the ‘positive’ test and instructions on the back of the packaging (Figure 6C). When Oxford-bound Beth (Felicity Jones) has a pregnancy scare in *Albatross* (2011), a coming-of-age story set in a family-run seaside hotel, streetwise Emilia (Jessica Brown Findlay) fetches her a test from the local pharmacy (Figure 6D). The friends embrace after a Clearblue Digital close-up reveals that Beth is ‘Not Pregnant’. Clearblue is shoplifted in *Now Is Good* (2012) and used by sixteen-year-old Kerrie (Sandra Enogie) in Lucia Yandoli's *Love Letter* (2012), an educational film made in collaboration with Highbury Grove School in Islington, North London (Figure 6E).

Beyond cinema and television, the Clearblue close-up has framed portrayals of pregnancy testing in other visual and narrative media. A sponge-tipped test on the cover of *Tough Choices: Young Women Talk about Pregnancy* (1999) evokes the first-hand accounts compiled within, and the three line-bearing tests pictured in Jools Oliver's *Minus Nine to One: The Diary of an Honest Mum* (2005) might be those taken ‘for [her] second baby, Daisy’, cherished ‘in a special box and, yes, the lines are still there just like magic!’ The ‘ghostly pale blue lines’ of a home pregnancy test remind a religious character in Zadie Smith's debut novel *White Teeth* (2000) of ‘the face of the madonna in the zucchini of an Italian housewife’, and on the first page of her fourth novel, *NW* (2012), the crisis posed by a positive result is conveyed in still fewer words, a literary close-up of sorts: ‘Blue cross on a white stick, clear, definitive. What to do?’^88^

**Figure 8.**
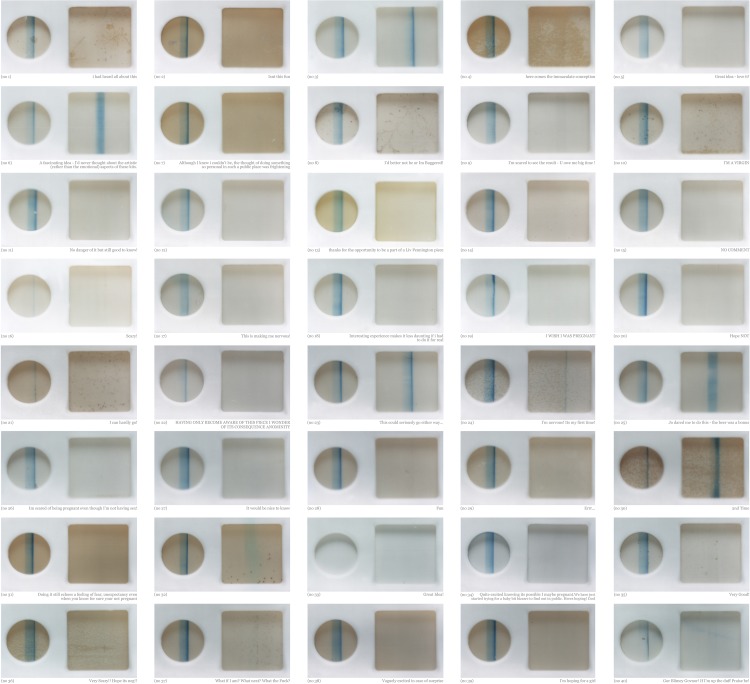
*Private View* (Liv Pennington, 2006), Digital C type on Aluminium, 80 × 76 cm. Courtesy of Liv Pennington.

From Josephine Pryde's Embryos and Estate Agents: L'Art de Vivre at the East End's Chisenhale Gallery (2011) to Home Truths: Photography, Motherhood and Identity at the Photographer's Gallery (2013–2014), photographic pregnancy tests now adorn London's leading galleries with some regularity.^89^ Love Is What You Want (2011), the Southbank Centre's Tracey Emin retrospective, displayed the artist's used pregnancy tests, now historical artefacts from a 1999 piece.^90^ London-based artist Gina Glover's *Yes!* uses rows of pregnancy tests as tally marks to ‘translate the experience of waiting for results during the process of IVF into a stylized, repetitive sentence of images ending in victory’.^91^ And Liv Pennington projected extreme close-ups of pregnancy test results ‘live in real-time’ at performances of *Private View* (2002–2010) in London, Poitiers, Oslo and Manchester. Comprising ‘forty different women's pregnancy tests from the London performance, combined with text written by the women as they were waiting to take their test’,^92^ the photographic companion piece makes apparent the fingerprint-like uniqueness of each result (Figure 8).^93^

Today, the moment of pregnancy testing is an extraordinary event of everyday life that most people will first encounter on screen – not only in cinema and on television, but increasingly also on laptops, tablets and phones. In January 2015, the *Daily Mail Online* reported that the keyword search for ‘pregnancy test results’ produced more than 18,000 YouTube videos.^94^ Breaking the cultural taboo on disclosing pregnancy before the first routine scan at twelve weeks, ‘WombTubers’ – who freely appropriate the Clearblue close-up – have created a virtual network of support and sympathy for women who experience miscarriage.^95^ But even when pregnancy testing is not filmed for public consumption, cinema and television occasionally provide the frame.^96^ For instance, in her intimate pregnancy memoir, *Expecting* (2016), Edinburgh journalist Chitra Ramaswamy imagines a ‘camera lens hovering above [her] head’ and allows herself a ‘Hitchcockian moment of suspense’, before finally indulging in the revelatory ‘close-up’ – a moment she compares to a ‘staunchly white’ television advert.^97^ So familiar are the dramatic conventions of the big and small screen that, at least for some women, pregnancy testing can feel a bit like being in a movie.

## The flexible politics of the close-up

From the Southbank Centre to WombTube, *EastEnders* to *NW*, the appropriation and aestheticization of pregnancy testing apparently knows no bounds. This article has sought to recover how the Clearblue close-up, a surprisingly little-researched cliché, was established and stabilized. Focusing on the intersection of technological, cultural and media change, it has attempted to explain the British explosion of televisual and cinematic pregnancy testing in terms of several conditions of possibility that converged in the late 1980s and early 1990s: the rapid commercial success of Clearblue, itself related to the biotech boom; NHS cutbacks; the rise of health consumerism; the media's dual obsession with teenage mothers and older career women trying to conceive; Channel 4's influence on dramatic television and cinema; ITV's restrictive attitude towards advertising; and Unipath's permissive attitude towards placement.

Free, no-strings-attached placement was especially crucial in Britain, where the non-commercial BBC controlled a large market share. Television and cinema's dramatic close-up and reaction shot, often of a young woman's distraught face, reached larger, younger and more diverse audiences than advertisements in women's magazines ever could. Constitutive of Britain's increasingly public culture of reproduction, this remarkably stable aesthetic convention did much to consolidate Clearblue as a recognizable brand. More than television ad campaigns, which were prohibitively expensive and highly regulated, it was the rise of socially engaged drama and embedded marketing that – prior to Clearblue Digital – helped equate the sight of thin blue lines with pregnancy.^98^ Though often implicating unprotected, underage and otherwise transgressive sex, the innocuous plastic stick also redirected viewers’ attention away from the messier aspects of conception.^99^ Recognizably branded packaging, written instructions and thin blue lines were dramatically charged, coded with meaning and acceptably abstract. The Clearblue close-up ambiguously concealed as much as it revealed. Its visual politics were sufficiently flexible for the aesthetic convention first established in television drama to be appropriated by filmmakers, artists and WombTubers. Abstraction, ambiguity and flexibility were its keys to success.

## Footnotes

1 But see Jesse Olszynko-Gryn, ‘Pregnancy testing in Britain, *c*.1900–67: laboratories, animals and demand from doctors, patients and consumers’, unpublished PhD thesis, University of Cambridge, 2014.

2 Sarah A. Leavitt, ‘“A private little revolution”: the home pregnancy test in American culture’, Bulletin of the History of Medicine (2006) 80, pp. 317–3451680986610.1353/bhm.2006.0064, 339. See further Leavitt's online exhibition, A Thin Blue Line: The History of the Pregnancy Test, at http://history.nih.gov/exhibits/thinblueline.

3 Lauren Rosenwarne, Periods in Pop Culture: Menstruation in Film and Television, Lanham: Lexington Books, 2012, pp. 180–184.

4 Douglas Holt and Douglas Cameron, Cultural Strategy: Using Innovative Ideologies to Build Breakthrough Brands, Oxford: Oxford University Press, 2012, pp. 202–219. On consumers’ perception of Clearblue Digital as the ‘Rolls Royce of pregnancy tests’ see Emily Jane Ross, ‘Exploring tentativeness: risk, uncertainty and ambiguity in first time pregnancy’, unpublished PhD thesis, University of Edinburgh, 2015, p. 120.

5 On 1980s Britain see Stephen Brooke, ‘Living in “new times”: historicizing 1980s Britain’, History Compass (2014), 12, pp. 20–32; Florence Sutcliffe-Braithwaite, ‘Observing the 80s’, Twentieth Century British History (2014), 25, pp. 484–495.

6 Michael Mulkay, The Embryo Research Debate: Science and the Politics of Reproduction, Cambridge: Cambridge University Press, 1997; Duncan Wilson, The Making of British Bioethics, Manchester: Manchester University Press, 201425320834.

7 Martin Durham, Sex and Politics: The Family and Morality in the Thatcher Years, Basingstoke: Macmillan Education, 1991; Sarah Franklin, Celia Lury and Jackie Stacey (eds.), Off-Centre: Feminism and Cultural Studies, London: HarperCollinsAcademic, 1991; Jane Pilcher, ‘Gillick and after: children and sex in the 1980s and 1990s’, in Jane Pilcher and Stephen Wagg (eds.), Thatcher's Children? Politics, Childhood and Society in the 1980s and 1990s, London: Falmer Press, 1996, pp. 77–93; Lisa Arai, Teenage Pregnancy: The Making and Unmaking of a Problem, Bristol: Policy, 2009.

8 On ovulation test kits see Chapter 4 of Jenna Healey, ‘Sooner or later: age, pregnancy, and the reproductive revolution in late twentieth-century America’, unpublished PhD thesis, Yale University, 2016.

9 See Patricia Day and Rudolf Klein, ‘The politics of modernization: Britain's National Health Service in the 1980s’, Milbank Quarterly (1989) 67, pp. 1–342509874; Soraya de Chadarevian, ‘The making of an entrepreneurial science: biotechnology in Britain, 1975–1995’, Isis (2011) 102, pp. 601–6332244854010.1086/663596; Alex Mold, Making the Patient–Consumer: Patient Organisations and Health Consumerism in Britain, Manchester: Manchester University Press, 2015. On the lucrative market for home diagnostic tests see Kenneth Green, ‘Shaping technologies and shaping markets: creating demand for biotechnology’, Technology Analysis & Strategic Management (1991) 3, pp. 57–76; Geoffrey Jones and Alison Kraft, ‘Corporate venturing: the origins of Unilever's pregnancy test’, Business History (2004) 46, pp. 100–122.

10 Simon Voss, ‘The usage of pregnancy tests in one health district’, British Journal of Obstetrics and Gynaecology (1992) 99, pp. 1000–1003147700210.1111/j.1471-0528.1992.tb13706.x.

11 Justin Smith and Paul McDonald (eds.), *Channel 4 and British Film Culture*, special issue of Journal of British Cinema and Television (2014) 11, pp. 413–516; Hannah Andrews, British Cinema: Convergence and Divergence since 1990, Houndmills: Palgrave MacMillan, 2014.

12 Raymond Williams, Television: Technology and Cultural Form, London: Fontana, 1974; Dana Polan, ‘Raymond Williams on Film’, Cinema Journal (2013) 52, pp. 1–18.

13 Film studies of the close-up have typically concentrated on the face. See, for example, Mary Ann Doane, ‘The close-up: scale and detail in the cinema’, differences (2003) 14, pp. 89–111; Hannah Landecker, ‘Cellular features: microcinematography and film theory’, Critical Inquiry (2005) 31, pp. 903–936; Béla Balázs, Early Film Theory: Visible Man and the Spirit of Film, New York: Berghahn, 2010, pp. 38–45, 100–111; Paul Coates, Screening the Face, Houndmills: Palgrave Macmillan, 2012.

14 Jesse Olszynko-Gryn, ‘The demand for pregnancy testing: the Aschheim–Zondek reaction, diagnostic versatility, and laboratory services in 1930s Britain’, Studies in History and Philosophy of Biological and Biomedical Sciences (2014) 47, pp. 233–2472438801410.1016/j.shpsc.2013.12.002PMC4275600.

15 Olszynko-Gryn, op. cit. (1), p. 15. See also Adrian Bingham, Family Newspapers? Sex, Private Life, and the British Popular Press 1918–1978, Oxford: Oxford University Press, 2009, p. 129.

16 John B. Gurdon and Nick Hopwood, ‘The introduction of *Xenopus laevis* into developmental biology: of empire, pregnancy testing and ribosomal genes’, International Journal of Developmental Biology (2000) 44, pp. 43–5010761846.

17 I have not been able to locate a copy of Stanford's film, which may well be lost, but traces of both films are abundant, for example, in the British Journal of Photography (1948) 95, p. 445; Proceedings of the Zoological Society of London (1948) 118, p. 539; and *The Year's Work in the Film*, 1949, p. 40, 1954, p. 54. See also the relevant entries in the BFI collection at http://collections-search.bfi.org.uk/web/Details/ChoiceFilmWorks/150120712; http://collections-search.bfi.org.uk/web/Details/ChoiceFilmWorks/150015227. Ciba, alongside Schering and Organon, controlled much of the market for sex hormones in the 1920s and 1930s. See Christina Ratmoko, Damit die Chemie stimmt: Die Anfänge der industriellen Herstellung von weiblichen und männlichen Sexualhormonen 1914–1938, Zurich: Chronos, 2010.

18 In the US, for example, a storyline about pregnancy testing with frogs was excised from the script of a major Hollywood production in 1944. See David A. Kirby, this issue.

19 Olszynko-Gryn, op. cit. (1), p. 119.

20 Joan Seaward, ‘Pregnancy test … a modern scientific achievement’, *Woman*, 24 June 1961, p. 27; Olszynko-Gryn, op. cit. (1), pp. 188–202. Marketed from 1950 to 1978 by the West German pharmaceutical company Schering, Primodos contained the same hormones as in the contraceptive pill. As with ‘the pill’, Primodos was also prescribed therapeutically as a treatment for irregular menstruation. When used diagnostically as a pregnancy test, Primodos was intended to induce menstrual bleeding in non-pregnant women only (a ‘negative’ result); no bleeding meant pregnancy (a ‘positive’ result). Primodos was significantly cheaper and more convenient than ordering a laboratory test and so it was widely prescribed, on the NHS, especially by overworked doctors in less affluent parts of the country.

21 Jesse Olszynko-Gryn ‘When pregnancy tests were toads: the *Xenopus* test in the early NHS’, Wellcome History (2013), 51, pp. 1–3.

22 Emma L. Jones, ‘Abortion in England, 1861–1967’, unpublished PhD thesis, Royal Holloway, University of London, 2007, p. 234.

23 See Barbara L. Brookes, Abortion in England, 1900–1967, London: Croom Helm, 1988; Lara V. Marks, Sexual Chemistry: A History of the Contraceptive Pill, New Haven, CT: Yale University Press, 2001; Jeffrey Weeks, Sex, Politics and Society: The Regulation of Sexuality since 1800, 3rd edn, London: Routledge, 2013, pp. 321–356; Lesley Hall, *Sex, Gender and Social Change in Britain since 1880*, 2nd edn, Houndmills: Palgrave Macmillan, pp. 148–164.

24 Delia Davin, ‘Using commercial pregnancy test in the 1960s’, email to author, 19 January 2014.

25 Olszynko-Gryn, op. cit. (1), p. 242.

26 Eric Schaefer, *Sex Scene: Media and the Sexual Revolution*, Durham, NC: Duke University Press; Nick Hopwood, Peter Murray Jones, Lauren Kassell and Jim Secord, ‘Introduction: communicating reproduction’, Bulletin of the History of Medicine (2015) 89, pp. 379–4042652166610.1353/bhm.2015.0064PMC4655582.

27 Samantha Lay, British Social Realism: From Documentary to Brit-Grit, London: Wallflower, 2002; Fran Bigman, ‘“Nature's a wily dame”: abortion in British literature and film, 1907–1967, unpublished PhD thesis, University of Cambridge, 2014.

28 Waris Hussein, email to author, 13 August 2014.

29 ‘Pregnancy testing: opposition by B.M.A.’, *Pharmaceutical Journal*, 20 September 1969, p. 319.

30 On *Women's Hour* and British radio's female listeners more generally see Jenni Murray, The Woman's Hour, London: BBC Books, 1996; Caroline Mitchell (ed.), Women and Radio: Airing Differences, London: Routledge, 2000, pp. 64–72; Maggie Andrews, Domesticating the Airwaves: Broadcasting, Domesticity and Femininity, London: Continuum, 2012. See also Jacqueline Kavanagh, ‘The BBC's written archives as a source for media history’, Media History (1999) 5, pp. 81–86.

31 Letter to Mrs Helen Graham, 9 November 1971, ‘Predictor home pregnancy test’, Wellcome Library, London (PP/GRA/B.4). Predictor was invented and patented in the late 1960s by New York graphic designer Meg Crane. See Pagan Kennedy, ‘Could women be trusted with their own pregnancy tests?’, *New York Times*, 29 July 2016, at www.nytimes.com/2016/07/31/opinion/sunday/could-women-be-trusted-with-their-own-pregnancy-tests.html?_r=0.

32 ‘Home pregnancy test kit’, *Pharmaceutical Journal*, 13 November 1971, p. 475.

33 *Sex with Health: The Which? Guide to Contraception, Abortion and Sex-Related Diseases*, London: Consumers’ Association, 1974, pp. 53–54. See further Jesse Olszynko-Gryn, ‘The feminist appropriation of pregnancy testing in 1970s Britain’, *Women's History Review*, forthcoming in 2018.

34 ‘Pregnancy testing’, *Which?*, February 1982, pp. 106–107.

35 Mary Irwin, ‘What women want on television: Doreen Stephens and BBC television programmes for women, 1953–1964’, Westminster Papers (2011) 8, pp. 99–122.

36 *Coronation Street*, Episode 3, 16 December 1960; Richard Dyer *et al.*, *Coronation Street*, London: BFI, 1981; John Caughie, ‘Television and serial fictions’, in David Glover and Scott McCracken (eds.), The Cambridge Companion to Popular Fiction, Cambridge: Cambridge University Press, 2012, pp. 50–67. Smoking in pregnancy emerged as a public-health concern and social problem only in the 1970s. See Laury Oaks, Smoking and Pregnancy: The Politics of Fetal Protection, New Brunswick, NJ: Rutgers University Press, 2001.

37 *Coronation Street*, Episode 1587, 31 March 1976.

38 See Tom Bromley, All in the Best Possible Taste: Growing Up Watching Telly in the 1980s, London: Simon & Schuster, 2010, p. 285.

39 On science, technology and medicine on British television see, for example, Ayesha Nathoo, Hearts Exposed: Transplants and the Media in 1960s Britain, Basingstoke: Palgrave Macmillan, 2009, pp. 86–110; Tim Boon, ‘“The televising of science is a process of television”: establishing *Horizon*, 1962–1967’, BJHS (2015) 48, pp. 87–1212583379910.1017/S0007087414000405.

40 See Greg Dyke, ‘The doctor's warning that was eight years on the way’, *The Guardian*, 6 June 1978, p. 9; Jesse Olszynko-Gryn, ‘Drug scandals and the media: the unresolved case of Primodos’, *The Guardian H Word*, 22 March 2017, at www.theguardian.com/science/the-h-word/2017/mar/22/drug-scandals-and-the-media-the-unresolved-case-of-primodos. The Ciba footage was again reused in a recent Sky News documentary, *Primodos: The Secret Drug Scandal*, now available on YouTube.

41 Peter Williams and Gordon Stevens, ‘What now for test tube babies?’, New Scientist (4 February 1982) 93(1291), pp. 311–31611655400.

42 On the origins of BPAS see David Paintin, Abortion Law Reform in Britain, 1964–2003: A Personal Account, Stratford: BPAS, 2015, pp. 66–74.

43 Jonathan Bignell and Stephen Lacey, ‘Introduction’, in Bignell and Lacey (eds.), British Television Drama: Past, Present and Future, 2nd edn, Basingstoke: Palgrave Macmillan, 2014, pp. 1–15. On the production of meaning and affect through close-up shots and editing see, for example, Stephen Prince and Wayne E. Hensley, ‘The Kuleshov effect: recreating the classic experiment’, Cinema Journal (1992) 31, pp. 59–75.

44 Christine Geraghty, ‘British soaps in the 1980s’, in Dominic Strinati and Stephan Wagg (eds.), Come On Down? Popular Media Culture in Post-war Britain, London: Routledge, 1992, pp. 133–149; Geraghty, ‘Social issues and realist soaps: a study of British soaps in the 1980s/1990s’, in Robert C. Allen (ed.), To Be Continued … Soap Operas around the World, New York: Routledge, 1995, pp. 66–80.

45 Phil Redmond, ‘*Brookside*: the technology backstory’, in Bignell and Lacey, *British Television Drama*, op. cit. (43), pp. 62–69, 65.

46 Redmond, op. cit. (45), p. 67.

47 Dorothy Hobson, Channel 4: The Early Years and the Jeremy Isaacs Legacy, London: I.B. Tauris, 2008, pp. 37, 41.

48 Christine Geraghty, ‘*Brookside*: no common ground’, Screen (1983) 24, pp. 137–41; Lez Cooke, British Television Drama: A History, 2nd edn, London: Palgrave/BFI, 2015, pp. 164–165.

49 David Buckingham, Public Secrets: *EastEnders* and Its Audience, London: BFI, 1987, p. 1.

50 Buckingham, op. cit. (49), p. 16.

51 Buckingham, op. cit. (49), p. 1.

52 Buckingham, op. cit. (49), p. 14.

53 Buckingham, op. cit. (49), p. 25.

54 W. Stephen Gilbert, ‘Programme notes’, *The Listener*, 18 February 1988, p. 27.

55 Buckingham, op. cit. (49), p. 85.

56 Buckingham, op. cit. (49), p. 149.

57 Hilary Kingsley, ‘Good girls do get pregnant too!’, *Daily Mirror*, 23 September 1985, p. 9. Though taken off the market in 1978, Primodos may have been responsible for the rumour that pregnancy tests caused abortions.

58 See Rebecca Feasey, Mothers on Mothers: Maternal Readings of Popular Television, Bern: Peter Lang, 2016, pp. 81–82.

59 Documentaries include *Schoolgirl Mums* (Granada, 1989); *Sonia's Baby* (ITV, 1990); *Frankenstein's Baby* (ITV, 1990); and *Teenage Diaries* (BBC2, 1993).

60 See Lyn Thomas, ‘In love with *Inspector Morse*: feminist subculture and quality television’, Feminist Review (1995) 51, pp. 1–25.

61 Clive Cookson, ‘Two new babies on the way’, *Financial Times*, 7 July 1988, p. 13.

62 *Positively Negative* was O'Keefe's last role. The talented young actor best known for her performance in *Threads* (1984), the iconic BBC television drama about nuclear war, tragically died in a car accident shortly after shooting. On literacy in pregnancy and the readability (or not) of home test instructions see Uta Papen, ‘Pregnancy starts with a literacy event: pregnancy and antenatal care as textually mediated experiences’, Ethnography (2008) 9, pp. 377–402; Lorraine S. Wallace, Nikki B. Zite and Virginia J. Homewood, ‘Making sense of home pregnancy test instructions’, Journal of Women's Health (2009) 18, pp. 363–36810.1089/jwh.2008.098519243273.

63 Celia Ford, ‘Biotechnology products start to roll’, Unilever Magazine (1985) 57, pp. 10–11. See also ‘Monoclonal pregnancy test’, *New Scientist* (30 May 1985) 106(1458), p. 22.

64 Ford, op. cit. (63). Advertising campaigns, as a Carter Wallace marketing executive later explained, did not need to target women who dreaded pregnancy because they would ‘buy tests anyway’. Companies promoting ovulation kits also preferred to emphasize the planning of pregnancy rather than its avoidance. See Sue Platt, ‘Testing times on TV’, *Community Pharmacy*, 1 November 1995, pp. 12–14.

65 Lindsay Cook, ‘How the cuts cause a pregnant pause’, *The Guardian*, 4 November 1985, p. 21.

66 Ford, op. cit. (63).

67 Cookson, op. cit. (61). See further Virginia Berrige and Kelly Loughlin, ‘Smoking and the new health education in Britain 1950s–1970s’, American Journal of Public Health (2005) 95, pp. 956–9641591481610.2105/AJPH.2004.037887PMC1449291; Rosemary Elliot, Women and Smoking since 1890, London: Routledge, 2008; Salim Al-Gailani, ‘Pregnancy, pathology and public morals: making antenatal care in early twentieth-century Edinburgh’, in Janet Greenlees and Linda Bryder (eds.), Western Maternity and Medicine, 1880–1990, London: Pickering and Chatto, 2013, pp. 31–46.

68 ‘After Clearblue comes Clearplan’, Unilever Magazine (1987) 65, pp. 7–8.

69 ‘Clearblue one step by Unipath Limited’, Design Council Archive, Brighton (GB 1837 DES/DCA/30/7/1989/5/1).

70 Cookson, op. cit. (61); ‘New pregnancy test cuts out the chemistry lesson’, *New Scientist* (21 July 1988) 119(1622), p. 39.

71 Cookson, op. cit. (61).

72 Linda Conway, ‘Frontier of medical diagnostics’, Unilever Magazine (1989) 72, pp. 35–39.

73 Karen Ross, Black and White Media: Black Images in Popular Film and Television, Cambridge: Polity Press, 1996, p. 47.

74 Farah's subsequent discovery of a letter from Maryam's gynaecologist confirming her pregnancy irritated at least one critic, who complained that ‘pregnancy test kits featured confusingly in all the bedrooms’: Farrah Anwar, review of *My Sister Wife*, *Sight & Sound* (May 1992) 2(1), pp. 62–63, 63.

75 Gargi Bhattacharyya and John Gabriel, ‘Gurinder Chadha and the *Apna* generation: black British film in the 1990s’, Third Text (1994) 8, pp. 55–63.

76 Andrea Stuart, ‘Blackpool illumination’, Sight & Sound (February 1994) 4(2), pp. 26–27, 26.

77 Meera Syal, quoted in an email from Emma Paterson to the author, 24 August 2015.

78 Chris Hackley and Rungpaka Amy Hackley née Tiwsakul, ‘Observations: unpaid product placement’, International Journal of Advertising (2012) 31, pp. 703–718, 708. See further Rungpaka Amy Tiwsakul, Chris Hackley and Isabelle Szmigin, ‘Explicit, non-integrated product placement in British television programmes’, International Journal of Advertising (2005) 24, pp. 95–111.

79 Graeme Atkinson, email to the author, 20 August 2015. See also Sarah Ryle, ‘When is a prop not a prop? When it's a plug’, *The Observer*, 12 August 2001, p. 33.

80 Kerry Segrave, Product Placement in Hollywood Films: A History, Jefferson, NC: McFarland, 2004.

81 Platt, op. cit. (64), p. 12.

82 Platt, op. cit. (64), p. 12.

83 In the US in 1997 David Lynch directed a fifteen-second television commercial for Clearblue Easy One Minute pregnancy test as part of a $7 million campaign. Leavitt, op. cit. (2), p. 333.

84 Hannah Andrews, Television and British Cinema: Convergence and Divergence since 1990, Basingstoke: Palgrave Macmillan, 2014, pp. 75–80.

85 James Leggott, Contemporary British Cinema: From Heritage to Horror, London: Wallflower Press, 2008, pp. 68–71; Robert Murphy, ‘Citylife: urban fairy tales in late 90s British cinema’, in Murphy (ed.), *The British Cinema Book*, 2nd edn, London: BFI, pp. 296–300; Nigel Mather, Tears of Laughter: Comedy–Drama in 1990s British Cinema, Manchester: Manchester University Press, 2006.

86 Peter Matthews, review of *Sliding Doors*, *Sight & Sound* (June 1998) 8(6), pp. 55–56.

87 See Cecily Devereux, ‘“Chosen representatives in the field of shagging”: Bridget Jones, Britishness, and reproductive futurism’, Genre (2013) 46, pp. 213–237.

88 Zadie Smith, White Teeth, London: Hamish Hamilton, 2000, p. 515; Smith, NW, London: Hamish Hamilton, 2012, p. 1. For an early discussion of the ‘literary close-up’ see Gerald F. Noxon, ‘The anatomy of the close-up: some literary origins in the works of Flaubert, Huysmans and Proust’, Journal of the Society of Cinematologists (1961) 1, pp. 1–24.

89 See Rebecca Baillie, ‘Exhibition review: Home Truths: Photography and Motherhood’, Studies in the Maternal (2013) 5, pp. 1–9. See further Sandra Matthews and Laura Wexler, Pregnant Pictures, London: Routledge, 2000.

90 See Outi Remes, ‘Replaying the old stereotypes into an artistic role: the case of Tracey Emin’, Women's History Review (2009) 18, pp. 559–575.

91 Sarah Franklin, Biological Relatives: IVF, Stem Cells, and the Future of Kinship, Durham, NC: Duke University Press, 2013, p. 287.

92 See the artist's professional website at http://happyswappy.co.uk/portfolio/private-view-2002-2006.

93 *Private View* now hangs in the Salford University Midwifery Department as part of Birth Rites, the ‘first and only collection of contemporary artwork dedicated to the subject of childbirth’. See http://birthritescollection.org.uk.

94 Jill Foster, ‘Thought there was nothing left to shock you in this age of over-sharing? Women who put their pregnancy test videos on YouTube’, *Daily Mail Online*, 8 January 2015, at www.dailymail.co.uk/femail/article-2901288/Thought-left-shock-age-oversharing-Women-pregnancy-test-videos-YouTube.html. For an introduction to YouTube see Jean Burgess and Joshua Green, *YouTube: Online Video and Participatory Culture*, Cambridge: Polity, 2009; Michael Strangelove, *Watching YouTube: Extraordinary Videos by Ordinary People*, Toronto: University of Toronto Press, 2010.

95 See Linda L. Layne, Motherhood Lost: A Feminist Account of Pregnancy Loss in America, New York: Routledge, 2003.

96 See, for example, Robert Kubey and Mihaly Csikszentmihalyi, Television and the Quality of Life: How Viewing Shapes Everyday Experience, Hillsdale, NJ: Lawrence Erlbaum Associates, 1990.

97 Chitra Ramaswamy, Expecting: The Inner Life of Pregnancy, Glasgow: Saraband, 2016, pp. 4–5. Some women liken the twelve-week ultrasound scan to ‘watching a television programme’. See Ross, op. cit. (4), p. 173.

98 The phrase ‘thin blue line’ has acquired other meanings beyond Clearblue pregnancy tests, namely the association with law enforcement conveyed in *The Thin Blue Line* (Errol Morris, 1988). Massive Attack's debut studio album *Blue Lines* was released in 1991, around the time Clearblue was taking off, but is probably not a direct reference.

99 Notably dramatic close-ups of reproductive technologies in British cinema and television include a misplaced diaphragm in *Robin Redbreast*, a *Play for Today* about pagan ritual murder (10 December 1970); Durex condom packaging in *Rita, Sue and Bob Too* (Alan Clarke, 1987); and a turkey baster (for artificial insemination) in an infamous episode of *Brookside* (13 November 1997). Oral contraception features in *Educating Rita* (Lewis Gilbert, 1983), though not in close-up, and structures the entire plot of *Prudence and the Pill* (Fielder Cook and Ronald Neame, 1968). In contrast to pregnancy testing, however, these other products did not inspire tropes; not flexible enough to fit a wide range of narratives and ignored by embedded marketing, they may also have been too visually boring or too explicitly linked to sex.

